# The U-POCUS protocol: urinalysis and point-of-care ultrasound to exclude symptomatic ureterolithiasis in emergency department patients

**DOI:** 10.1007/s43678-025-00998-z

**Published:** 2025-09-13

**Authors:** Matthew Tripod, Kendra Mendez, Matthew Berger, Claire Shaffer, Timothy Yeung, Thomas G. Costantino, Steven Peterson, Ryan C. Gibbons

**Affiliations:** 1 Department of Emergency Medicine, Capital Health, Pennington, NJ USA; 2 Department of Emergency Medicine, Lewis Katz School of Medicine at Temple University, Philadelphia, PA USA; 3 Department of Emergency Medicine, University of Pittsburgh Medical Center – Harrisburg, Harrisburg, PA USA; 4 Department of Emergency Medicine, Loma Linda University, Loma Linda, CA USA; 5 Temple University, Philadelphia, PA USA

**Keywords:** Ureterolithiasis, Point-of-care ultrasound, Hematuria, Urinalysis, Urétéro-lithiase, échographie au point d’intervention, hématurie, analyse d’urine

## Abstract

**Objective:**

Urolithiasis is a common urological condition accounting for more than 1.3 million emergency department visits annually with costs exceeding $2.8 billion (Scales et al. in Eur Urol. 62:160–5, 2012;Eaton et al. in J Endourol 27:1535–1538, 2013;Antonelli et al. in Eur Urol 66:724–729, 2014;). Non-contrast computed tomography of the abdomen and pelvis remains the diagnostic gold standard. Studies assessing urinalysis and renal point-of-care ultrasound (PoCUS), individually, to diagnose symptomatic ureterolithiasis demonstrate inadequate sensitivities (Mefford et al. in West J Emerg Med 18:775, 2017;Eray et al. in Am J Emerg Med 21:152–4, 2003;Luchs et al. in Urology 59:839–842, 2002;Smith-Bindman et al. in N Engl J Med 371:1100–1110, 2014;Riddell et al. in West J Emerg Med 15:96–100, 2014;Rosen et al. in J Emerg Med 16:865–870, 1998;Gaspari and Horst in Acad Emerg Med 12:1180–1184, 2005;Watkins et al. in Emerg Med Australas 19:188–195, 2007;). The primary objective of this study was to assess the test characteristics of the U-PoCUS (urinalysis with renal point-of-care ultrasound) protocol.

**Methods:**

This was an Institutional Review Board approved, multi-center, retrospective chart review at a university-based healthcare system. Study investigators included all patients who presented from January 1, 2016 through June 30, 2020, and underwent computed tomography of the abdomen and pelvis and had a urinalysis and PoCUS for suspected ureterolithiasis. Investigators utilized MedCalc (Version 19.1.6) and standard 2 × 2 tables to calculate test characteristics with 95% confidence intervals (CI).

**Results:**

Study investigators enrolled 183 patients, including 122 patients diagnosed with computed tomography confirmed ureterolithiasis and 61 patients without it. The combination of hematuria and/or hydronephrosis on PoCUS had a sensitivity of 99.2% (95.6–100) and a specificity of 14.8% (7–26.2) for the presence of urolithiasis. Positive predictive value and negative predictive value were 69.9% (67.7–72.1) and 90% (53.9–98.6), respectively.

**Conclusion:**

The presence of hematuria and/or hydronephrosis was 99.2% sensitive for the presence of ureterolithiasis diagnosed on computed tomography of the abdomen and pelvis. The U-PoCUS protocol missed only one symptomatic ureterolithiasis.

## Résumé

**Objectifs:**

La lithiase urinaire est une affection urologique courante qui représente plus de 1,3 million de visites aux urgences chaque année avec des coûts dépassant 2,8 milliards de dollars [1-3]. La tomodensitométrie sans contraste de l’abdomen et du bassin reste la norme de référence pour le diagnostic. Les études évaluant l’analyse d’urine et l’échographie rénale au point de soins (PoCUS), individuellement, pour diagnostiquer une urétérolithiase symptomatique démontrent des sensibilités inadéquates [6-13]. L’objectif principal de cette étude était d’évaluer les caractéristiques du test du protocole U-PoCUS (analyse d’urine avec échographie rénale au point de soins).

**Méthodes:**

Il s’agissait d’une revue rétrospective multicentrique des dossiers approuvée par le Institutional Review Board dans un système de santé universitaire. Les chercheurs de l’étude ont inclus tous les patients qui se sont présentés entre le 1er janvier 2016 et le 30 juin 2020, qui ont subi une tomodensitométrie de l’abdomen et du bassin et qui ont subi une analyse d’urine et un PoCUS pour une uretérolithiase suspectée. Les chercheurs ont utilisé MedCalc (Version 19.1.6) et des tableaux standard 2x2 pour calculer les caractéristiques du test avec des intervalles de confiance à 95 %.

**Résultats:**

Les chercheurs de l’étude ont inclus 183 patients, dont 122 patients diagnostiqués avec une urétérolithiase confirmée par tomodensitométrie et 61 patients sans. La combinaison d’hématurie et/ou d’hydronéphrose sur PoCUS avait une sensibilité de 99,2 % (95,6-100) et une spécificité de 14,8 % (7-26,2) pour la présence de lithiase urinaire. La valeur prédictive positive et la valeur prédictive négative étaient de 69,9 % (67,7-72,1) et 90 % (53,9-98,6), respectivement.

**Conclusions:**

La présence d’une hématurie et/ou d’une hydronéphrose était sensible à 99,2 % pour la présence d’urétérolithiase diagnostiquée par tomodensitométrie de l’abdomen et du bassin. Le protocole U-PoCUS n’a manqué qu’une seule urétérolithiase symptomatique.

## Clinician’s capsule

**Table:** 

***What is known about the topic?***
Symptomatic ureterolithiasis is a common emergency medicine urologic condition with costs exceeding $2.8 billion.
***What did this study ask?***
Can the absence of hematuria and hydronephrosis exclude ureterolithiasis?
***What did this study find?***
The U-PoCUS Protocol has a sensitivity of 99% and missed only one symptomatic ureterolithiasis.
***Why does this study matter to clinicians?***
To expedite diagnoses and to reduce unnecessary computed tomography scans with its resultant cost and radiation exposure without affecting diagnostic accuracy.

## Introduction

Urolithiasis, including nephrolithiasis and ureterolithiasis, is a common urological condition accounting for more than 1.3 million emergency department visits annually with costs exceeding $2.8 billion. [1–3]. Non-contrast computed tomography of the abdomen and pelvis remains the diagnostic gold standard. However, non-contrast computed tomography of the abdomen and pelvis is costly, utilizes ionizing radiation, and increases patient length of stay. Notwithstanding, non-contrast computed tomography of the abdomen and pelvis use has risen considerably over the preceding years [4, 5].

Urinalysis and renal point-of-care ultrasound (PoCUS) offer a safer, more cost-effective means of diagnosing ureterolithiasis assessing the presence of hematuria and hydronephrosis, respectively. However, individually, each lacks adequate sensitivity for the diagnosis. Studies determined that urinalysis has a sensitivity between 70 and 90% for hematuria, and PoCUS has a sensitivity between 60 and 85% for hydronephrosis [6–13].

The incidence of urolithiasis has increased 70% in recent years, affecting nearly 10% of the population with a recurrence rate of 50% [2]. Furthermore, according to the Nationwide Emergency Department Sample, non-contrast computed tomography of the abdomen and pelvis utilization doubled between 2006 and 2015 [2]. By 2030, costs associated with urolithiasis are expected to exceed $5 billion [2, 14]. Despite its increased use, no studies have demonstrated improved patient benefit [15, 16]. In fact, recurrent radiation exposure increases cancer risk [17–19].

The primary objective of this study was to determine if the U-PoCUS (urinalysis with renal point-of-care ultrasound) protocol can exclude ureterolithiasis.

## Methods

### Study design and time period

The Temple University Institutional Review Board approved this multicenter, structured retrospective chart review of presentations between January 1, 2016 and June 30, 2020. Study authors followed the Kaji et al. and STARD guidelines and checklists for retrospective study designs assessing the accuracy of diagnostic tests [20, 21]. Study investigators received no funding.

### Study setting and population

Investigators conducted the study at an urban, university healthcare system with more than 200,000 annual adult and pediatric emergency department (ED) visits over three diverse clinical campuses: an urban, tertiary care academic medical center; an urban, academic community center; and a suburban, academic community center. Investigators enrolled all patients aged 18 years old or older, who presented to the ED with suspected urolithiasis and received a computed tomography of the abdomen and pelvis, renal PoCUS, and urinalysis. Exclusion criteria included: patients with a pre-existing ureteral stent.

Our health system credentials all Emergency Physicians in the core American College of Emergency Physicians point-of-care ultrasound applications [22].

### Intervention

A blinded (to the study objectives) investigator queried the electronic medical record, Medhost (Franklin, TN), to identify patients using the following search terms and International Classification of Diseases, Ninth Revision Diagnosis Codes: calculus or stone: kidney/nephrolithiasis (592.0), ureter (592.1), urinary unspecified (592.9), bladder (594.1), and ureteral (594.2); and renal colic (788.0), hematuria (599.7), back pain (724.5), flank pain (789.0). Patients meeting appropriate inclusion criteria underwent chart abstraction to obtain the following data: renal PoCUS, urinalysis and microscopy, point-of-care urine dipstick, and computed tomography of the abdomen and pelvis results. The data report is computer-generated, obviating the need for human abstraction of each individual medical record thereby reducing investigator bias and subjective interpretation of results.

### Outcome measures

Per the American Urological Association guidelines, investigators defined hematuria as the following: (1) more than 2 red blood cells per high power field or (2) any positive point-of-care urine dipstick if microscopy was not performed or unavailable [23]. If both results were available, investigators utilized the microscopy. Laboratory technicians interpreted all urinalyses and microscopies. Emergency medicine nursing staff performed all point-of-care urinalysis analyses. Study investigators did not report on the experience of nursing staff. Board-certified radiologists interpreted all computed tomography of the abdomen and pelvis studies.

An emergency medicine post-graduate year 1–3, emergency ultrasound fellow, or emergency medicine attending performed all renal PoCUS examinations, which involves transverse and longitudinal sweeps assessing for hydronephrosis within the collecting system. Physicians perform each renal PoCUS utilizing the low-frequency (2–5 MHz) curvilinear transducer in the abdominal setting on a GE Logiq E (Wauwatosa, WI) or SonoSite Edge or M-turbo (Bothell, WA). Study investigators considered any reported degree of hydronephrosis (mild, moderate, or severe) as diagnostic. In our Department of Emergency Medicine, we do not routinely assess the presence of ureteral stones or jets. Further, we did not account for the patient receiving intravenous fluid, which may affect the degree of hydronephrosis. Similarly, we did not document when the provider performed the renal PoCUS, specifically pre- or post-void. Bladder overdistension may result in reflux and hydronephrosis. Likewise, we did not account for if the provider had imaging or laboratory results prior to the PoCUS examination.

Prior to starting their internship, our emergency medicine residents participated in an introductory four-hour introduction to PoCUS course taught by our emergency ultrasound faculty. Additionally, each resident completes a three-week emergency ultrasound rotation during their internship in accordance with Accreditation Council for Graduate Medical Education and American College of Emergency Physicians guidelines [22, 24].

The primary outcome of this study was to assess the test characteristics of the U-PoCUS protocol. Study investigators utilized computed tomography of the abdomen and pelvis as the diagnostic reference standard. Secondary outcomes included the test characteristics of PoCUS and urinalysis, individually; the accuracy of hydronephrosis on PoCUS compared to computed tomography of the abdomen and pelvis; and the accuracy of point-of-care urinalysis compared to microscopy.

### Data analysis and sample size

Previous data suggest up to 30% of patients with ureterolithiasis will not have microscopic hematuria [6–8]. Moreover, PoCUS has a limited sensitivity between 60 and 85% for detecting hydronephrosis [9–13]. Investigators hypothesized a sensitivity of 75% for the U-PoCUS protocol. As mentioned previously, the sensitivity of non-contrast computed tomography of the abdomen and pelvis exceeds 95% for the diagnosis of ureterolithiasis [11, 25–27]. Using this data and a power analysis of 80% with an alpha of 0.05, investigators calculated a sample size calculation of 98 patients diagnosed with ureterolithiasis to demonstrate a hypothesized 20% difference in sensitivities between non-contrast computed tomography of the abdomen and pelvis and the U-PoCUS protocol. Investigators report continuous and categorical data as means or medians with interquartile ranges or proportions with 95% confidence intervals (CIs) and utilized standard 2 × 2 tables on MedCalc (Version 19.1.6) to calculate test characteristics with 95% confidence intervals (CI).

## Results

**Fig. 1:**
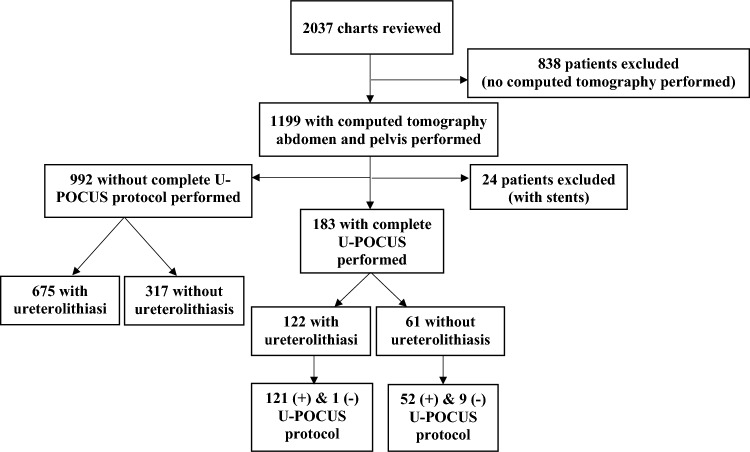
Patient flow chart. U-POCUS Protocol, Urinalysis + renal point-of-care ultrasound

Study investigators queried 2073 patient records, excluding 838 patients for lack of computed tomography of the abdomen and pelvis, 24 for pre-existing ureteral stents, and 992 patients for lack of a complete U-PoCUS protocol. Investigators included 183 patients; 122 patients diagnosed with computed tomography of the abdomen and pelvis confirmed ureterolithiasis and 61 patients without it. Figure 1 illustrates the patient flow chart, and Table 1 depicts the patients’ characteristics. Table 2 reviews the test characteristics for the protocol and each component individually.

**Table 1:** Patient Characteristics

Characteristic	U-POCUS Protocol (*n* = 183)	Without Complete U-POCUS Protocol (*n* = 992)
Age, Median (IQR), years	44 (21)	43 (23)
Sex, *N* (%)		
Female	97 (53)	575 (58)
Male	86 (47)	417 (42)
Chief complaint, *N* (%)		
Abdominal pain	44 (24)	327 (33)
Back pain	33 (18)	148 (15)
Flank pain	88 (48)	407 (41)
Hematuria	9 (5)	59 (6)

*IQR* Interquartile range. U-POCUS Protocol Urinalysis and renal point-of-care ultrasound

**Table 2:** Test Characteristics of Protocol and Individual Components vs Computed Tomography of the Abdomen and Pelvis

	Urinalysis &/or Renal point-of-care ultrasound (95% CI)	Urinalysis (95% CI)	Renal point-of-care ultrasound (95% CI)
Sensitivity	99.2 (95.6–100)	90.2 (83.5–94.8)	74.6 (65.9–82)
Specificity	14.8 (7–26.2)	26.2 (15.8–39.1)	62.3 (49–74.4)
Positive likelihood ratio	1.2 (1.1–1.3)	1.2 (1.0–1.4)	2 (1.4–2.8)
Negative likelihood ratio	0.1 (0–0.4)	0.4 (0.2–0.7)	0.4 (0.3–0.6)
Positive predictive value	69.9 (67.7–72.1)	71 (67.6–74.2)	79.8 (73.8–84.7)
Negative predictive value	90 (53.9–98.6)	57.1 (40.3–72.5)	55.1 (46.1–63.8)
Accuracy	71 (63.9–77.5)	68.9 (61.6–75.5)	70.5 (63.3–77)

*CI* Confidence interval

The combination of hematuria and/or hydronephrosis on PoCUS had a sensitivity of 99.2% (95.6–100) and a specificity of 14.8% (7–26.2) for the presence of urolithiasis. Positive predictive value and negative predictive value were 69.9% (67.7–72.1) and 90% (53.9–98.6), respectively. Overall, the protocol had an accuracy of 71% (63.9–77.5) with positive and negative likelihood ratios of 1.2 (1.1–1.3) and 0.1 (0–0.4), respectively. Nonetheless, only 1 of 122 patients (< 0.01%) with computed tomography of the abdomen and pelvis proven ureterolithiasis had neither hematuria on urinalysis nor hydronephrosis on renal PoCUS.

Table 3 reviews test characteristics of PoCUS for the diagnosis of hydronephrosis compared to computed tomography of the abdomen and pelvis. PoCUS missed 26 cases of hydronephrosis, yielding a sensitivity of 78.2 (69.7–85.2). This reflects previous literatures [10–13]. Notably, 22 patients had mild or “minimal or slight” hydronephrosis while three had moderate hydronephrosis on computed tomography of the abdomen and pelvis. One computed tomography of the abdomen and pelvis interpretation did not report the degree of hydronephrosis.

**Table 3:** Test Characteristics of Point-of-Care Ultrasound for Hydronephrosis Compared to Computed Tomography of the Abdomen and Pelvis

	Renal Point-of-Care Ultrasound (95% CI)
Sensitivity	78.2 (69.7–85.2)
Specificity	65.6 (52.7–77.1)
Positive likelihood ratio	2.3 (1.6–3.2)
Negative likelihood ratio	0.3 (0.2–0.5)
Disease prevalence	65 (57.6–71.9)
Positive predictive value	80.9 (72.5–87.6)
Negative predictive value	61.8 (49.2–73.3)
Accuracy	73.8 (66.8–80)

*CI* Confidence interval

Table 4 delineates the test characteristics of point-of-care urinalysis compared to microscopy as the gold standard. In 120 of 133 (90%) cases, the microscopies and point-of-care urinalysis had equivalent results. In eight cases, the point-of-care urinalysis differed from the microscopy performed in the laboratory. Nurses interpreted two of the point-of-care urinalyses as negative with [1–3 red blood cells] and [3–5 red blood cells] noted in the laboratory. One of those had a ureteral stone with hydronephrosis present on PoCUS. Five cases had small blood on point-of-care urinalysis with three having [0–2 red blood cells] and two having [none seen red blood cells] present on microscopy. 55 patients had point-of-care urinalysis only.

**Table 4:** Test Characteristics Point-of-Care Urinalysis vs Urinalysis with Microscopy

	Point-of-care urinalysis (*n* = 133; 95% CI)
Sensitivity	98 (92.8–99.8)
Specificity	78.6 (59.1–91.7)
Positive likelihood ratio	4.6 (2.3–9.3)
Negative likelihood ratio	0.03 (0.1–0.1)
Disease prevalence	77.8 (69.5–84.7)
Positive predictive value	94.1 (87.6–97.8)
Negative predictive value	91.7 (73–99)
Accuracy	93.7 (87.9–97.2)

*CI* Confidence interval

## Discussion

### Interpretation of findings

**Fig. 2:**
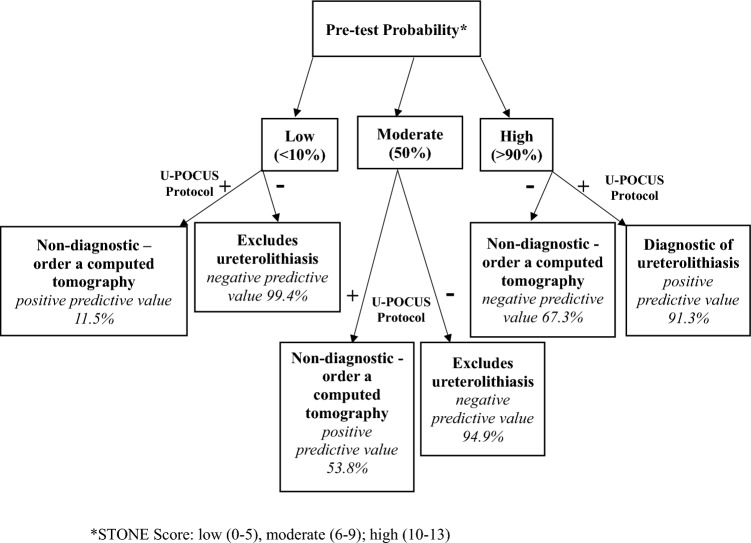
Clinical decision flow chart. U-POCUS Protocol, Urinalysis + renal point-of-care ultrasound

Taken in isolation without assessing prevalence and pre-test probabilities, the protocol’s accuracy, positive and negative predictive values, and positive and negative likelihood rations do not alter the management of patients with suspected ureterolithiasis. However, if we utilize the STONE score: low (0–5), moderate (6–9), high (10–13) to estimate pre-test probabilities and then apply the U-PoCUS results, the resultant positive and negative predictive values allow providers to reduce computed tomography use by either diagnosing or excluding ureterolithiasis in certain high or low risk patients. Each score has a corresponding prevalence of ureterolithiasis, low < 10%, moderate 50%, and high > 90%. For a patient with a low score, the negative predictive value is 99.4%, which excludes ureterolithiasis. For a high score patient, the positive predictive value is 91.3%, which is diagnostic. A moderate score yields a positive predictive value of 53.8% and a negative predictive value of 94.9%. In these cases, we defer to the provider's gestalt. Figure 2 provides a suggested clinical decision flow chart utilizing the protocol based on pre-test probabilities and their corresponding positive and negative predictive values.

### Comparison to previous studies

In a large, multi-center trial, Smith-Bindman et al. showed that a PoCUS-first approach for evaluating ureterolithiasis had similar outcomes to non-contrast computed tomography and radiology-performed ultrasound, while reducing radiation exposure [9]. Notably, fewer than 10% of patients require urologic intervention [28], questioning the routine use of computed tomography. The U-PoCUS protocol aims to exclude symptomatic ureterolithiasis and reduce radiation, missing only one case in our cohort (sensitivity > 99%).

Individually, urinalysis and PoCUS lack sufficient sensitivity. Up to 30% of patients may not have microscopic hematuria, particularly with higher degrees of hydronephrosis due to complete ureteral obstruction. PoCUS shows 60–80% sensitivity for detecting hydronephrosis [6–13]. Our findings support combining both modalities to improve diagnostic accuracy. While the absence of ureteral jets has been studied, it does not predict hospitalization or outcomes [29], so our ED does not assess them routinely. Likewise, clinical decision tools like the STONE and STONE PLUS scores have attempted risk stratification but remain unreliable [30–32].

### Strengths and limitations

This study was appropriately powered for statistical significance and included a diverse group of clinical providers with variable PoCUS experience. Results suggest the protocol can reduce unnecessary radiation, cost, and diagnostic delays without sacrificing sensitivity. However, it is limited by its retrospective design and convenience sampling, leading to selection bias and a smaller sample size. Likewise, we did account for site enrollment creating a sampling bias; nor could we control for missing or incorrect data. We excluded patients suspected of urolithiasis who did not undergo computed tomography, i.e., those diagnosed clinically or with urinalysis and/or PoCUS, removing a significant portion of the initial cohort. Moreover, we included patients diagnosed with contrast computed tomography, which is not standard of care. Furthermore, non-contrast and contrast computed tomography are both imperfect gold standards with ~ 95% sensitivities [11, 25–27].

Reliance on emergency physicians’ subjective PoCUS interpretation reduces validity, and we lacked quality assurance data to verify findings, including hydronephrosis degree. Additionally, we did not assess providers’ PoCUS experience or whether they had knowledge of preceding imaging and laboratory results, or even intravenous fluid administration timing; all are potential confounders [33]. Our department’s robust ultrasound division, training, and resources limit generalizability, including resident-performed PoCUS. Using point-of-care dipsticks when microscopy was unavailable and not accounting for nursing experience with dipstick interpretation limit credibility. Nonetheless, point-of-care urinalysis aligned with standard results in 97% of cases. Ninety percent of our patients had microscopic hematuria, reflecting current literature that 10–30% may lack hematuria [6–8]. However, PoCUS may be more sensitive in patients with hematuria, possibly inflating our results [12]. Finally, we did account for menses in female patients, which may have further increased our protocol’s sensitivity.

### Clinical implications

Our results suggest that the absence of both hematuria and hydronephrosis excludes symptomatic ureterolithiasis, obviating the need for non-contrast computed tomography of the abdomen and pelvis. Likewise, a positive protocol in patients with high pre-test probability is diagnostic of ureterolithiasis, reducing the need for non-contrast computed tomography of the abdomen and pelvis, again apart from complicated febrile urinary tract infections. Our results are promising with respect to expediting diagnoses and treatment. Moreover, the U-PoCUS protocol has the potential to minimize unnecessary radiation as well as patient length of stay and cost.

### Research implications

While these data are promising, future larger, multi-center, prospective studies are necessary to further validate the U-PoCUS protocol. Given our interest focused solely on the diagnostic accuracy of the protocol, we did not assess patient-centered outcomes. Future prospective studies should evaluate not only validate our findings but assess the clinical impact of the U-PoCUS protocol as well, including patient management, outcomes, and length of stay; misdiagnoses; reduction in both computed tomography use and cost; and return visits.

## Conclusion

The U-PoCUS protocol has a sensitivity of 99% and missed only one symptomatic ureterolithiasis. By utilizing the U-POCUS protocol with respect to corresponding pre-test probabilities, providers can expedite the diagnosis of symptomatic ureterolithiasis as well as safely exclude it while minimizing unnecessary radiation and diagnostic delays.
